# Uncovering Virus-Virus Interactions by Unifying Approaches and Harnessing High-Throughput Tools

**DOI:** 10.1128/mSystems.00121-19

**Published:** 2019-06-04

**Authors:** Samuel L. Díaz-Muñoz

**Affiliations:** a Department of Microbiology and Molecular Genetics, University of California, Davis, Davis, California, USA; b Genome Center, University of California, Davis, Davis, California, USA

**Keywords:** coinfection, ecology, evolution, reassortment, sociovirology

## Abstract

Virus-host interactions have received much attention in virology. Virus-virus interactions can occur when >1 virus infects a host and can be deemed social when one virus affects the fitness of another virus, as in the well-known case of superinfection exclusion. Coinfection and subsequent social interactions can change viral pathogenicity, host range, and genetic composition, with implications for human health and viral evolution. I propose that this field can be advanced by bringing new perspectives into virology (e.g., social evolution theory) and uniting disciplinary divides within virology (classical, host-focused, and ecoevolutionary). The development of novel high-throughput tools that meld molecular and evolutionary approaches can harness viral diversity as an experimental asset to understand complex viral social interactions. A greater knowledge of virus-virus interactions will lead to the reformulation of basic concepts of virology and advances in applied virology, with new treatments that harness interactions between viruses to fight viral and bacterial infections.

## Abstract (precis)

Virus-host interactions have received much attention in virology. Virus-virus interactions can occur when >1 virus infects a host and can be deemed social when one virus affects the fitness of another virus, as in the well-known case of superinfection exclusion.

## PERSPECTIVE

**FIG 1:**
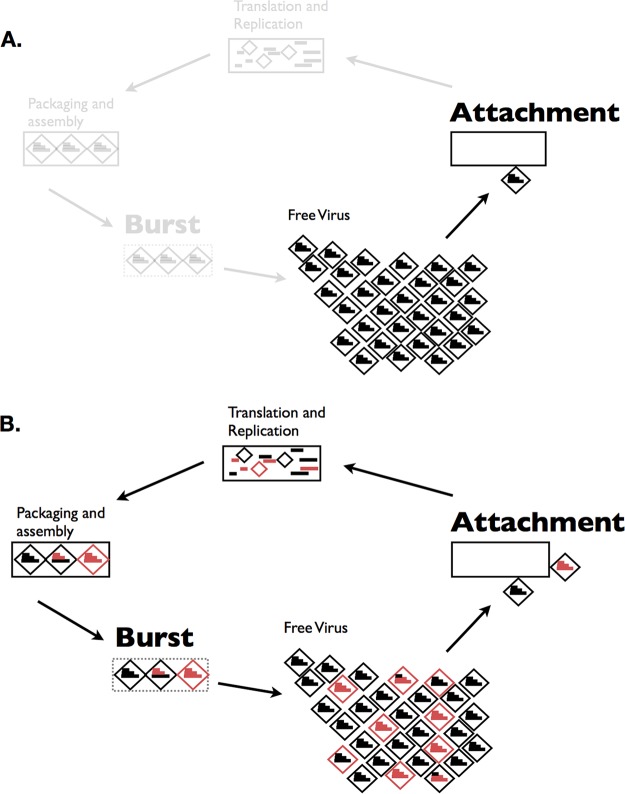
Coinfection can change every stage of the viral life cycle. (A) Example of a simplified viral life cycle of a generic segmented virus. The bright portion of the figure emphasizes how many textbook depictions of viral life cycles end with the production of many virions, but start with just one virion. (B) A revision of the viral life cycle when starting from a coinfection of two viruses. Different colors of the genome segments are intended to show potential interactions between the viruses at different points of the life cycle.

Virology, etymologically, means the study of viruses. However, much of the discipline has studied viruses as disease agents, often focusing on a single infectious strain and host pairing (1). Perhaps the best illustrations of this predisposition are textbook depictions of viral life cycles, which start with one virus infecting a cell, replicating many copies of itself, only to resume the cycle with just one virus (Fig. 1A). Yet, more than one virus can be present in the host (coinfection), providing the opportunity for viruses to interact. When these interactions affect the fitness of another coinfecting virus, this is, in Darwinian terms, a social interaction. For instance, infection with one virus can positively or negatively (e.g., superinfection exclusion) affect an infection with a second virus and these interactions can involve similar or heterologous viruses (2). The era of investigation of the microbiome and genomics in biology has made apparent that viral coinfections are exceedingly common (3–6), setting the stage for viral social interactions. Furthermore, a growing body of evidence shows that coinfections cause increased pathogenicity, host jumps, and genetic exchange, all with implications for human health and viral evolution. If our models are revised to account for coinfection, the potential for virus-virus interactions becomes apparent at virtually at every step of the cycle (Fig. 1B).

In contrast to the potential importance of virus-virus interactions, we still are in the beginning stages of knowing the frequency, mechanisms, and consequences of these interactions. Four questions are the key to determining how virus-virus interactions affect the viral life cycle and to developing fundamental principles of viral social interactions: (i) What is the extent of coinfection and are there specific ecological correlates or molecular mechanisms that promote coinfection?; (ii) What is the frequency of evolved cooperation and cheating and how are they manifested mechanistically?; (iii) To what extent can viral proteins be shared among viruses, representing potential public goods?; and (iv) Are virus-virus interactions context dependent and what are their consequences across scales (cell, host, populations), especially in natural settings? Below, I suggest two approaches to make progress towards answering these questions and highlight studies from our group and many colleagues that are advancing the field of virus-virus interactions.

## UNITING PERSPECTIVES AND APPROACHES IN VIROLOGY

Studies on virus-virus interactions have been conducted for a long time, with examples ranging from early phage research (7) to studies of satellite viruses and defective interfering particles. However, the results of these and subsequent studies have not been consolidated into a field that generates core concepts that are carried over more broadly into virology. To highlight the contributions of studies of virus-virus interactions and encourage more collaborative research, a group of us has advocated for this emergent field, which we have termed “sociovirology” (2, 8, 9). We have proposed harnessing a rich body of social evolution research to explain virus-virus interactions (2), as has been done for bacteria (10) and for other levels of biological organization. To further build community and collaboration, at the invitation of Asher Leeks, I have helped to co-organize a symposium to exchange ideas at the upcoming American Society for Microbiology Microbe 2019 meeting.

These efforts are designed to blur the sharp disciplinary boundary between “classical virology” (focused on viruses as disease agents) and virus studies that test evolutionary or ecological concepts (11). A potential resolution for this divide is to recognize viruses as organisms (12) which are under selection to optimize various aspects of their life cycle, including the host, but also virus-virus interactions and virus-environment interactions. An organismal approach can help to integrate these disciplines, providing an enormous opportunity to meld conceptual and technical approaches from classical virology and viral ecology and evolution. Recent studies illustrate this approach, uncovering that, across diverse viral taxa, vesicles provide a specific molecular mechanism to transmit multiple viral particles together, with the group of particles serving as the infectious unit (13–15). These landmark studies provide an answer to one of the key sociovirology questions: the extent of coinfection and whether there are specific molecular mechanisms mediating coinfection. In doing so, these studies have shaken one of the conceptual pillars of virology, i.e., the notion that the virus particle serves as the infectious unit.

The opportunity of unifying these approaches is accompanied by a technical challenge. How do we adapt the tools of classical virology to dissect the molecular mechanisms contributing to virus-virus interactions in more than just a few laboratory strains? Conversely, how do we develop additional experimental approaches applicable to the diversity of strains that evolutionary and ecological virology has uncovered in nature? Based on experiences in our lab, I suggest that high-throughput tools provide an opportunity to use viral diversity as an experimental asset to unify approaches and uncover viral social interactions.

## HIGH-THROUGHPUT BIOLOGY TO UNCOVER VIRAL SOCIAL INTERACTIONS

Virus-virus interactions represent an inherently complex area of virology in that they involve, at a minimum, two or more viruses. Furthermore, these interactions can change due to the ecological setting, molecular mechanisms, and population processes within and between hosts. For instance, cooperation between variants of influenza virus A were detected in cell culture, but not in human clinical samples (16). A second example comes from viral genetic exchange. Our work has provided evidence of substantial strain-to-strain variation in reassortment in RNA phages, which can be linked to the geographic origin of strains (17), counter to prevailing wisdom in the field (18). Studies of HIV have likewise revealed complex interactions among a diverse viral population in basic science studies (19) and clinical analyses (20, 21). High-throughput tools will be essential to study these complex social phenotypes with diverse strains. However, it is unlikely that “off-the-shelf” sequencing approaches will be appropriate. The use of high-throughput tools in virology is typically polarized, with the tools used either to conduct wide surveys across many taxa or strains with sequencing or to perform detailed experimental laboratory studies focused on a single strain using sequencing (22) or single-cell microfluidics (23). Creative use of currently available tools can bridge the gap between surveys and experiments by allowing parallel experimentation with diverse strains, uncovering complex phenotypes by using viral diversity as an asset. These applications of high-throughput tools must be accompanied by a solid theoretical foundation that allows hypothesis testing or model validation. This is of course true with any biological study, but is particularly relevant for studies of virus-virus interactions because so little is known. While a broadly accepted corpus of theoretical research in virus-virus interactions is lacking, recent studies have been developing theory to answer some key sociovirology questions regarding the frequency and mechanisms of cooperation (24) and the consequences of virus-virus interactions to within host-virus diversity (25) and to integrate theoretical approaches that remain separate in the literature, despite their equivalence (26).

A deliberate approach is needed to connect data from biological findings made at different scales (11). The same way that single-cell patterns of innate immunity are important, but can have varying consequences for groups of cells, virus-virus interactions have patterns at the single-cell level that may have very different outcomes according to population structure. A recent study nicely illustrates this point by connecting viral complementation during coinfection with the spatial structure of the virus-host population to explain the maintenance of incomplete influenza A virus particles (27). Ultimately, these approaches can be formalized, leading to an emergent systems biology of virus-virus interactions that will mathematically relate findings at multiple scales from single cells to ecosystems. The challenges to reaching this goal are formidable. Systems biology of virus-host interactions is already a complex endeavor, but by using the right tools, theoretical approach, and study systems, significant headway will be made in understanding the social lives of viruses.

## FUTURE DIRECTIONS

Over the next 5 years, I anticipate five major changes in the emergent field of sociovirology. First, high-throughput single-cell and subcellular techniques will yield unprecedented resolution on viruses’ interactions within their hosts. Second, viral surveys will establish the landscape for coinfection in the environment and within hosts. Third, experimental studies will be driven by theory and mathematical modeling, increasingly developing a body of work that establishes general principles of virus-virus interactions and stimulating new developments in social evolution theory. Fourth, social-evolution-based treatments (e.g., the use of defective viruses as antivirals and optimized phage therapy cocktails) that work orthogonally to current approaches will move from the proof-of-concept stage to testing and development. Finally, these advances will collectively conspire to bring virologists of all stripes together to establish principles of virus-virus interactions, integrate them seamlessly into virology, and communicate their importance to the broader microbiological, biological, and medical communities. This future will bring a reformulation of basic concepts of virology and lead to advances in applied virology, with new treatments that harness interactions between viruses to treatments of viral and bacterial infections.
